# Digging for historical data on the occurrence of benthic macrofaunal species in the southeastern Mediterranean

**DOI:** 10.3897/BDJ.4.e10071

**Published:** 2016-11-01

**Authors:** Irini Tsikopoulou, Aglaia Legaki, Panagiotis D. Dimitriou, Evangelia Avramidou, Nicolas Bailly, Stamatina Nikolopoulou

**Affiliations:** ‡ University of Crete, Heraklion, Greece; § University of Ahtens, Athens, Greece; | Aristotle University of Thessaloniki, Thessaloniki, Greece; ¶ Institute of Marine Biology, Biotechnology and Aquaculture, Hellenic Centre for Marine Research (HCMR), Heraklion, Greece

**Keywords:** marine biodiversity, macrofauna, Egypt expedition, eastern Mediterranean, historical dataset, data digitization, data rescue

## Abstract

**Background:**

The benthic macrofaunal biodiversity of the southeastern Mediterranean is considerably understudied compared to other Mediterranean regions. Monitoring biodiversity in this area is crucial as this region is particularly susceptible to biological invasions and temperature alteration. Historical biodiversity data could provide a useful baseline for monitoring potential changes and provide informarion to support a better understanding of the possible effects of anthropogenic activities on marine benthic communities.

**New information:**

In this study, performed under the LifeWatchGreece Research Infrastructure, we present historical benthic occurrence data obtained from the sampling expedition carried out in 1933 by Adolf Steuer in the coastal area around Alexandria, Egypt, eastern Mediterranean. The occurrences were geo-referenced to more than 170 stations, mostly located in the area of Alexandria, and the nearby coasts and lakes. All records were digitized and species names were cross-checked and taxonomically updated using the World Register of Marine Species. The outcome clearly shows that such initiatives can reveal an unexpected amount of highly valuable biodiversity information for “data-poor” regions.

## Introduction

At the beginning of the 20th century, the importance of recording marine biodiversity was already recognized. Numerous expeditions had been organized with the aim of investigating “local fauna and flora” in various areas of the world. In 1924, Cambridge Expedition at the Suez Canal recorded the fauna of the Red Sea (Fox 1926), while Danish Oceanographical Expedition in 1908-1910 provided biological and hydrographical information for the Mediterranean and Adjacent Seas (Schmidt 1912). During these scientific expeditions, local biodiversity of various taxonomic groups was collected, recorded and the outcome was published in many scientific volumes. These historical occurrence data could provide a useful baseline for monitoring potential alterations, although they are often fragmented and found only in hard copy and grey literature. Such information is invaluable and needs to be digitized as it can provide the historical context for present observations and facilitate the process of setting correct reference conditions (Borja et al. 2012); it can also support predictive modeling of the consequences of human activities for the environment and biodiversity (Costello et al. 2013a). Additionally, historical datasets often contain descriptions of new species that are important for taxonomy as the first description of a species has legal priority for the name of this species (Costello et al. 2013b).

In this study, we present occurrence data which were digitized from 14 publications on the Egypt Expedition under the general report "The fishery grounds near Alexandria" made by Adolf Steuer and his colleagues and published between 1935-1940. Twelve of these publications included occurrence data on twelve macrofaunal groups and two of them were preliminary reports which described the sampling protocols that were followed during the expedition (Table 1). The digitization of "The fishery grounds near Alexandria" - Egypt Expedition - is a part of a broader strategy for the LifeWatchGreece Research Infrastructure, which aims at the digitization of historical datasets that contain biodiversity information from the Mediterranean region.

## Project description

### Title

Digitization of historical dataset - Egypt Expedition

This dataset combines the benthic macrofaunal data obtained from the floristical and faunistical survey of A. Steuer in the coasts near Alexandria in 1933. The results of this expedition were published in 38 volumes between 1934 and 1940. In this study, we present the digitized data of 14 volumes concerning macrofaunal taxa (1935-1940). In rare cases, occurrence data for planktonic species were available in these volumes and were included in the digitized datasets.

### Personnel

The datasets were digitized by the LifeWatchGreece data management team. Irini Tsikopoulou (data manager), Stamatina Nikolopoulou (data, database and webgis application manager) and Aglaia Legaki (data manager) were the resource creators, Panagiotis D. Dimitriou (data manager) and Evangelia Avramidou (data manager) were content providers. Nicolas Bailly has checked difficult taxonomic cases.

The original data were collected by Dr. Adolf Steuer, professor at the University of Innsbruck, who organized and led the sampling expedition to the coasts near Alexandria, Egypt. After sampling, all collected specimens were preserved and sent to several experts for taxonomic identification. Each expert was responsible for the publication of his macrofaunal report.

### Study area description

**Figure 1.**
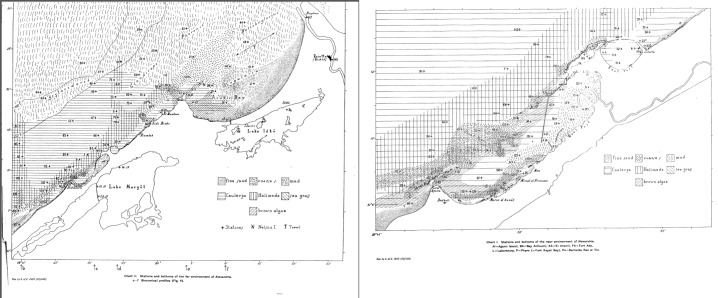
Stations as they were mapped in Steuer's preliminary report (1935).

The study area of the Egypt Expedition is located between the Western and Eastern harbors of Alexandria, including nearby localities such as Abukir Bay, the Suez Canal and the lakes Edku and Mariout (Fig. 1). The majority of the sampling stations do not exceed the isobath of 200 meter. The coasts that were investigated were in part shallow and sandy, in part steep. Information concerning the sediment characteristics and vegetation of the studied area was also available and included in the digitized dataset.

### Design description

Data rescue/digitization

### Funding

The digitization of this historical dataset was supported by the LifeWatchGreece Research Infrastructure, funded by the Greek Government under the General Secretariat of Research and Technology (GSRT), ESFRI Projects, Structural Funds, OPCE II.

Regarding the Egypt expedition, the Egyptian Ministry of Finance funded the whole sampling campaign and provided Dr Steuer with the expedition vehicle of the Marine Laboratory of Alexandria, a 15 m long motor boat named “El Hoot”. Additionally, the Ministry of Finance provided access to the Marine Laboratory of Alexandria located within the harbor area of the Alexandria city, as well as a small row boat, an automobile and the sampling equipment.

## Sampling methods

### Sampling description

Sampling took place at 172 locations in the marine area off Alexandria, in the Suez Canal, in the Nile river and in two lagoons (Lake Mariout and Lake Edku). Adolf Steuer was in charge of the sampling which lasted from April to November of 1933. A motor-launch (small military vessel) 15 m long, named "El Hoot", belonging to the Marine Laboratory, was used for the one-day trips at sea. Since it was difficult to sail too far from the shore, only two stations (station 26 and station 64) surpassed the 200 m isobath. In some cases a small rowing boat was also used. The collection of benthic samples was done almost exclusively by using a dredge with an opening of 20x70 cm. In only one case sampling was performed with a large otter trawl (bottom trawling) in the eastern part of Bay of Abukir, at a depth of 20 meters. A bottom sampler (Petersen's grab) of 0.2 m^2^ surface was also used once in the Eastern harbor due to difficulties in its manipulation (Vatova 1935). In shallow water, where no other equipment could be used, the samples of benthos were taken by diving. The sites where the sampling was performed along the coast were: the mouth of the Nile near Rosetta (Rashid), Lake Mariout and Lake Edku. Concerning planktonic samples, vertical hauls were operated using a medium sized net with buckets of celluloid with a gauge bottom.

### Quality control

Every single dataset was digitized manually from scanned documents. Some publications were in French or in German, depending on the author, and therefore the information was translated to English. Species names and sampling location names in the digitized datasets were kept same as in the original paper. Afterwards, all scientific names were cross-checked and taxonomically updated using the Taxon Match tool of the World Register of Marine Species (WoRMS) (WoRMS Editorial Board 2016). Station coordinates were produced by georeferencing maps from Fauvel (1937) using a Geographic Information System (GIS). The digitized datasets are presented in a standardised way, using Darwin Core terminology, informations on taxonomy, locality, sampling date, sampling protocol and individual measurements where they were available.

### Step description

**Digitization process**

The digitization of the historical publications concerning the Egypt Expedition is a challenging process due to their complexity and the variety of the format across the different faunistic reports. Information on the sampling protocol and the sampling sites were digitized mainly based on the preliminary reports of Steuer (1935) and Vatova (1935) enriched with information from maps and the main text in the rest of the publications. Occurrence data were digitized based on the individual faunistic reports. The data digitization was made using the Darwin Core terminology.

The digitization process of the Egypt Expedition datasets included several steps that are described below:

1. Data managers read and comprehended individual faunistic publications, in order to overcome difficulties originating from the heterogeneity in the format and the content among the historical papers. The original authors did not follow a specific format for the presentation of their results. Some of them included species distribution maps, some reported species list, sampling dates and depths, while others also recorded individual species counts. If there was a species list in the historical papers, species were recorded according to their taxonomical classification. Respectively, if there was a station list, stations were reported chronologically.
2. A spreadsheet was created for each faunistic report and were populated with original species names found at each location. In this stage of digitization process, obvious typographic errors were corrected. The spreadsheets also contained information on the sampling depth (minimum and maximum depth), sampling date (year, month, day), sampling protocol and habitat (substrate type and vegetation). For benthic samples, station depth and sampling depth were matched. In some faunistic reports, station depths were given in fathoms (i.e. approximately 1.8 meters). In these cases, station depths in the datasets were converted to meters. For some taxonomic groups, additional information such as sex, lifestage, individual counts or body length measurements were available, either on a species level or on a specimen level. Accepted taxon names and taxonomic classification, as derived from the World Register of Marine species, were also included in the spreadsheets.
3. After the digitization of all available information contained in the main text and tables in the publications, sampling stations were georeferenced using the species distribution maps in every faunistic report. Since there were no stations coordinates, latitude, longitude and coordinates uncertainty were estimated using a GIS based on the distribution maps in each publication and in Fauvel (1937). In cases, where a station was only referred to as a specific locality in the text, and not accompanied by a symbol on a map, a new station with higher uncertainty was created based on the locality description.
4. In the next step, a code (fieldNumber) was created for each sampling event. A unique event was defined as a sampling event that took place in a specific station at a specific time and sampling depth using a specific sampling protocol. In some cases several samples had been taken in a location without defining the sampling station but only the wider area. To represent these, a new station ID was created, accompanied by an respective location remark. A code (occurrenceID) was also created for each species occurrence record.
5. The outcome of the above digitization steps was twelve spreadsheets with 56 columns containing occurrence data of twelve benthic macrofaunal taxa. These tables were combined in the MedOBIS PostgreSQL database in order to correct mistakes originating from differences in the information or absence of information derived from Steuer's preliminary report (1935) and individual faunistic reports. In cases of corrections, original information was always kept as a remark in the dataset.
6. The final step was the publication of the data through the MedOBIS Data Repository (MedOBIS IPT - http://ipt.medobis.eu/) and the MedOBIS Geoportal (http://medobis.portal.lifewatchgreece.eu/viewer). The Integrated Publishing Toolkit (IPT) (Wieczorek and Braak 2015, http://www.gbif.org/ipt) is a free open source software tool and is used to publish and share biodiversity datasets through the GBIF network (http://www.gbif.org/). It uses Darwin Core (http://rs.tdwg.org/dwc/terms/) and Ecological Metadata Language (EML) standards. All datasets are distributed as Darwin Core Archives, a compressed file that contains: one or more data files with details for event, occurrence and measurements in a comma-separated or tab-separated list, an archive descriptor (meta.xml) file describing the individual data file columns used and a metadata file on EML describing the entire dataset. The datasets concerning different taxonomic groups were published separately with their metadata and individual URLs (Table 2). Since the repository does not currently support species measurements such as body and carapace length, this information is added in the supplementary files of the present paper (Suppl. material 1), following the recently developed Environmental data and Event schema of OBIS (De Pooter et al., in prep). The datasets are also available on MedOBIS Geoportal (a virtual lab on LifeWatchGreece Research Infrastructure), where all the taxonomic groups are visualized on a map. The Geoportal allows users to search and download marine species datasets from all over the Mediterranean in several different formats (CSV, KML, WFS, WMS). In addition, a MedOBIS mobile application has been developed on playstore (https://play.google.com/store/apps/details?id=com.Hcmr.LifeWatch).

**Difficulties regarding data digitization**

During the digitization process, several issues with the data were encountered. The majority of these problems were similar across datasets. In the following paragraphs, we will highlight the most common ones and explain how they have been dealt with.

1. Data on the same sampling event were scattered and repeated in different publications. This created inconsistencies both across and within publications. Information on stations characteristics and sampling protocol were often repeated with small differences, missings or typographic errors due to different languages in the publications as well as in the preliminary reports. Within each faunistic report, occurrence records were often presented in two different ways, once as a list of species by station and again as a list of stations by species, leading to small differences or typographic errors. For practical reasons, we decided to consider as correct the information on sampling protocol that was obtained from the preliminary reports and the information on species distribution obtained from species list rather than station list. In any case, different information was always kept as a remark in the datasets.
2. The final number of stations recorded was 172: a total of 150 benthic stations reported in the Steuer’s preliminary report (1935) enriched with 10 planktonic stations derived from the 12 faunistic reports and with 10 new stations generated during the digitization process. Some stations described only verbally in the faunistic reports were not on a map. For example, some species referred to be collected from the “eastern harbour, on the body of a ship” or “eastern harbour, epifauna” without displaying on the map. In such case, a new station was created (e.g. easterharbour1), in order to include all available information. Other examples were LacMarioutCenter and westernharbour1 stations. In addition, new station IDs had to be created by the data management team because some stations were reported in the historical maps without a station name. For example, a sampling position “near the bath” was mentioned and mapped in many reports without a specific station name. Other stations without a station name were coastAbuQir-nearRosetta, LakeEdku_marinebeach, LakeEdkubridge, offSidiBishr and Silsila.
3. Besides general difficulties, described above, some sampling stations needed extra consideration.
  - For station D2, the sampling date was not recorded in mollusks report (Steuer 1939b). Nevertheless, this gap was corrected using the 1st of October 1933 (1/10/1933) as the sampling date because all the trips were one day trips and in all papers D2 was visited only on that day.
  - Another problematic station was station 104. This station was reported in four faunistic reports: two of them without sampling date and the rest with different dates, 1/11/1933 in Sipuncula and 8/11/1933 in Polychaeta. Eventually, the date 8/11/1933 was considered as correct instead of 1/11/1933 and used for all the reports. This decision was made, because, as mentioned above, stations were reported in chronological order and station 104 was reported under the ones of 7/11 and before the ones of 9/11.
  - Some stations were reported with subdivisions. One of them was station 105 that was divided into 105a and 105b in some faunistic reports, but they were not mapped separately. In this case, we used the coordinates of station 105 with higher uncertainty for both subdivisions. In the report on mollusks, and specifically regarding the species *Cerithium vulgatum* and *Tricolia pullus*, the stations 105a and 105b were displayed on two maps in opposite locations (Steuer 1939b, figures 5 and 8 in pp.15 and 24).
  - Station 13 was also referred as 13b in the report on mollusks (Steuer 1939b) but it was mapped as station 13 and as a result we used the coordinates of station 13.
4. In some planktonic hauls it was difficult to distinguish sampling and station depth. For example, “…over about 15 fathoms 3 vertical hauls out of depths of 20 m” was the description of station plankton4a in the Sipuncula report (Steuer 1939a). Thus, it was assumed, in accordance with preliminary reports, that station depth was 15 fathoms (about 28 m) and sampling depth was 0-20 m.
5. In a few cases, specimens that were reported in the historical publications could not be taxonomically updated due to missing species name authorship or due to ambiguous names. For example, *Heliacus moniliferus* was recorded in the Mollusca report (Steuer 1939b), but actually this name is used for a fossil, not a living animal as reported in Steuer (1939b).
6. Original sampling date and sampling depth were not recorded in the historical papers concerning Echinodermata and Ascidiacea. During the digitization of all the datasets concerning the Egypt Expedition, it was clear that in most of the cases, sampling dates and sampling depths were the same as in other datasets of Egypt Expedition and as a result date and depth information were taken from other faunistic reports and the preliminary report.
7. Finally, maps were only available as a .pdf in the preliminary report so they were converted to a .jpg or .tiff, in order to proceed to integration in GIS and be georeferenced. Maps recorded the geographical information of the sampling stations. Maps were not ideally suited for georeferencing: they were not accurate, had no scale or map projection system, and the accuracy of the scanner which had created the digitized version was unknown. The original map had to be aligned with their actual geographical location, by linking each point to its equivalent on a modern, accurate digital map (Rumsey and Williams 2002). In the end, Geographic Coordinate System: GCS_WGS_1984 and Datum: D_WGS_1984 was defined as the geographical coordinate system for the maps and the stations were digitized in a new feature class in GIS on a scale of 1:250.000. However it is impossible to perfectly align old maps due to landscape changes over the decades. For example, both the harbor of Alexandria as well as Lake Mariout were significantly extended over the course of the 20th century. A coordinates uncertainty of around 200 meters was estimated by the root mean square (RMS) - this is the georeference error. This value describes how consistent the transformation is between the different control points. In some cases, where the sampling stations were not well defined on the maps (lack of station name, landscape changes) new points were placed on the map manually, with higher uncertainty. The planktonic stations were also placed manually on the map.

## Geographic coverage

### Description

**Figure 2.**
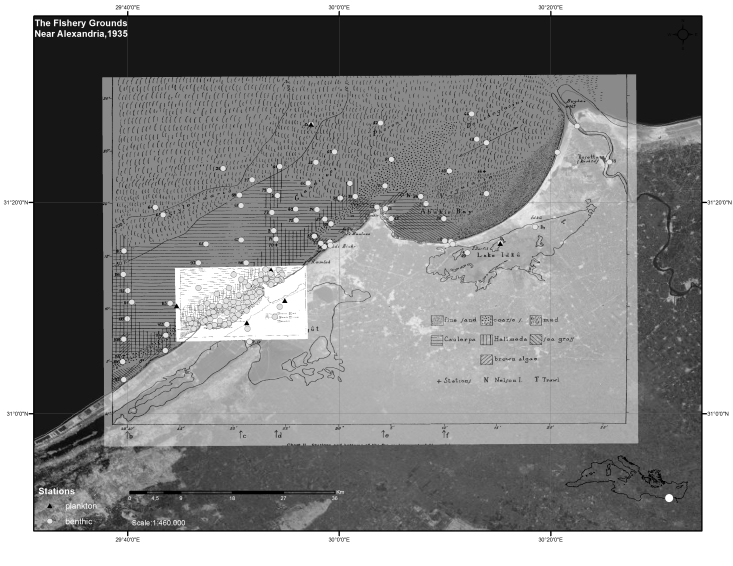
Georeferenced map of all stations from "The fishery grounds near Alexandria" macrofaunal reports.

The Egypt expedition covered, with 162 benthic and 10 planktonic stations, the area along the coasts of Alexandria, the Suez Canal, the Nile river and the lakes Edku and Mariout (Fig. 2).

### Coordinates

31.054 and 31.473 Latitude; 30.426 and 29.658 Longitude.

## Taxonomic coverage

### Description

**Figure 3.**
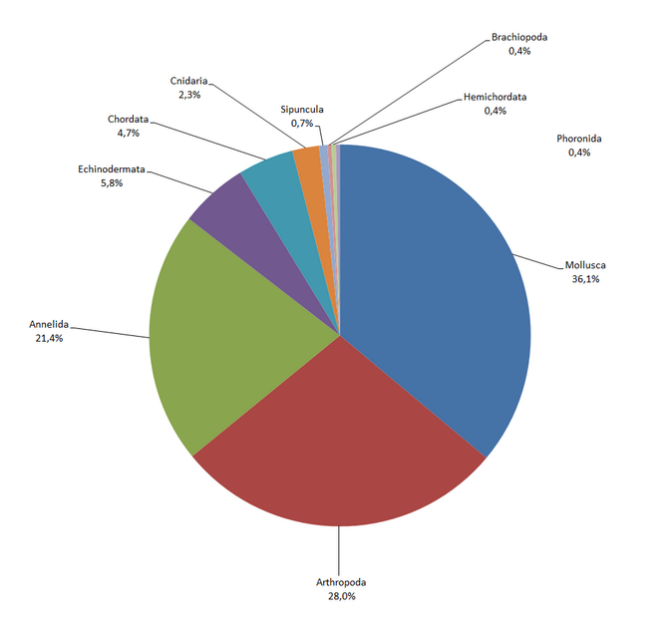
Percentage of macrofaunal species per phylum.

**Figure 4.**
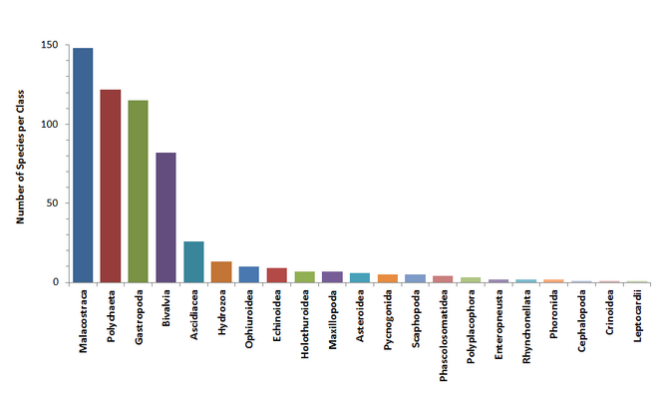
Number of macrofaunal species per class.

This set of historical data includes distribution information for 571 marine macrobenthic species belonging to 10 phyla, 21 classes and 257 families (Fig. 3). Malacostraca was the most speciose class with 26% of total species found, followed by Polychaeta (21%), Gastropoda (20%) and Bivalvia (14%) (Fig. 4). The family with the highest number of species richness was Syllidae (17 species), followed by Trochidae (14 species) and Veneridae (11 species). For the rest of the families, more than half of them (146 of 257) were represented by a single species.

These macrofaunal species were distributed in 172 stations located in the marine area off Alexandria, Egypt (Table 2). Species richness at the different sampling stations was very heterogenous. The most species rich stations were station 61, located above the isobath of 50 fathoms (90m), station 35, off Sidi Bishr and station 7 located close to the Eastern Harbour of Alexandria. The ten most common species in the study area are presented in Table 3. These species were found in more than 10% of the total number of stations.

### Taxa included

**Table:** 

Rank	Scientific Name	
phylum	Annelida	
phylum	Mollusca	
phylum	Arthropoda	
class	Hydrozoa	
phylum	Echinodermata	
phylum	Sipuncula	
class	Ascidiacea	
phylum	Phoronida	
phylum	Brachiopoda	
class	Enteropneusta	

## Temporal coverage

**Data range:** 1933 4 01 – 1933 11 18.

### Notes

Sampling started at April 1st 1933 and ended at November 18th 1933.

## Usage rights

### Use license

Creative Commons Public Domain Waiver (CC-Zero)

## Data resources

### Data package title

Digitization of historical dataset - Egypt Expedition﻿

### Number of data sets

1

### Data set 1.

#### Data set name

The fishery grounds near Alexandria

#### Data format

Text

#### Number of columns

1

#### Character set

UTF-8

#### Download URL

http://ipt.medobis.eu/

#### Description

This publication consists of 12 individual datasets containing different taxonomic groups as published by the original authors between 1935 and 1940 and 2 metadata only (Vatova 1935, Steuer 1935). All datasets are available via the IPT (http://ipt.medobis.eu/), serving as the Mediterranean node of the Ocean Biogeographic Information System (MedOBIS). Datasets will also be available on OBIS website in winter 2016. Individual download URLs for each dataset are available in Table 1. Table 4, Table 5 and Table 6 describe events, occurrences and measurements or facts, respectively.

## Additional information

**Conclusions**

Data rescue is an increasing need with expected effects on the scientific and societal perception of biodiversity. Despite the many challenges encountered during the digitization process of historical datasets (e.g. taxonomic updates, georeferencing, misspellings of taxa and places, compiling overlapping information from different publications), the outcome clearly shows that such initiatives are invaluable in making accessible previously unavailable biodiversity data.

Concerning the Egypt expedition, this paper is the first step for the digitization of the whole set of publications from "The fishery grounds near Alexandria". In Eastern Mediterranean, these data could be used to set the reference conditions for checking the invasion of alien species through the Suez Canal or to compare past species occurrences with current ones. In addition, the availability of these historical data through public databases (such as LifewatchGreece Research Infrastructure and MedOBIS) provides useful tools for present observations or monitoring potential change in benthic communities. Through virtual labs, scientists or other users could search, visualize on a map, combine and download species occurrences from all over the Mediterranean in several different formats.

Digitizing historical datasets offers also valuable information on functional species traits, as they usually contain individual characteristics, such as maturity and body length, and habitat characteristics, such as sediment type and vegetation. Information on functional species traits is required in describing species patterns and assessing future evolution of benthic communities.

## Supplementary Material

Supplementary material 1 Extended Measurement or Facts Data type: Measurements
Brief description: Body length measurements are available for the following datasets:
1. EgyptExpeditionCumaceaStomatopodaLeptostacaea
2. EgyptExpeditionPolychaeta
3. EgyptExpeditionAmphipoda
4. EgyptExpeditionTanaidaceaIsopoda
5. EgyptExpeditionMollusca
Carapace length measurements are available for the dataset EgyptExpeditionDecapoda
File: oo_94157.csv Irini Tsikopoulou, Stamatina Nikolopoulou, Aglaia Legaki

## Figures and Tables

**Table 1.** Taxa and related published volumes and information included in "The fishery grounds near Alexandria" dataset

**Taxon name in original dataset**	**Accepted Taxon name**	**Volume**	**Author**	**Date**	**Title**	**Download URL**
-	-	8	A.Steuer	1935	I. - Preliminary report	http://ipt.medobis.eu/resource?r=egyptexpeditionpreliminaryrepor
-	-	9	A. Vatova	1935	II. A bottom sample taken at Alexandria	http://ipt.medobis.eu/resource?r=egyptexpeditionbottomsampler
Cirripeds	Cirripedia	10	H. Broch	1935	III. - Cirrepeds	http://ipt.medobis.eu/resource?r=egyptexpeditioncirripeds
Hydroidea	Hydrozoa	13	A. Billard	1936	VI. - Hydroidea	http://ipt.medobis.eu/resource?r=egyptexpeditionhydroidea
Decapoda	Decapoda	15	H. Balss	1936	VII. Decapoda	http://ipt.medobis.eu/resource?r=egyptexpeditiondecapoda
Pantopoda	Pantopoda	16	H. Helfer	1936	VIII. - Pantopoda	http://ipt.medobis.eu/resource?r=egyptexpeditionpantopoda
Amphipoda	Amphipoda	18	A. Schellenberg	1936	X. - Amphipoda Benthonica	http://ipt.medobis.eu/resource?r=egyptexpeditionamphipoda
Annelides Polychetes	Polychaeta	19	P. Fauvel	1937	XI. - Annelides Polychetes	http://ipt.medobis.eu/resource?r=egyptexpeditionpolychaeta
Echinoderma	Echinodermata	21	Th. Mortensen & Ad. Steuer	1937	XIII. - Echinoderma	http://ipt.medobis.eu/resource?r=egyptexpeditionechinoderma
Cumacea, Stomatopoda, Leptostraca	Cumacea, Stomatopoda, Leptostraca	26	A. Steuer	1938	XVI. Cumacea, stomatopoda, leptostraca	http://ipt.medobis.eu/resource?r=egyptexpeditioncumaceastomatopodaleptostacaea
Ascidiacea	Ascidiacea	28	H. Harant	1939	XVii. Ascidiacea	http://ipt.medobis.eu/resource?r=egyptexpeditionascidiacea
Sipunculoidea, Phoronidea, Brachiopoda, Enteropneusta, Arcania	Sipuncula, Phoronida, Brachiopoda, Enteropneusta, Arcania	30	A. Steuer	1939	XVIII. - Sipunculoidea, Phoronidea, Brachiopoda, Enteropneusta and Arcania	http://ipt.medobis.eu/resource?r=egyptexpeditionsipunculoideaphoronidaebrachiopodaenteropneustaacrania
Mollusca	Mollusca	33	A. Steuer	1939	XIX. - Mollusca	http://ipt.medobis.eu/resource?r=egyptexpeditionmollusca
Tanaidacea, Isopoda	Tanaidacea, Isopoda	35	H. J. Larwood	1940	XXI. Tanaidacea and Isopoda	http://ipt.medobis.eu/resource?r=egyptexpeditiontanaidaceaisopoda

**Table 2.** List of sampling stations, their locality, habitat characteristics and species richness

**a/a**	**Station Name**	**Locality**	**Latitude**	**Longitude**	**Coordinates uncertainty**	**Habitat**	**Number of species**	**Number of phyla**
1	1	North of Abu Qir Bay	31,363663	30,016892	200	mud and stones, *Halimeda*	19	4
2	2	North of Pharo, Alexandria	31,288853	29,896681	200	*Halimeda*, sand, mud	35	4
3	3	North of Pharo, Alexandria	31,344246	29,902678	200	muddy, *Halimeda*, *Caulerpa*	22	6
4	4	Eastern Harbour, Alexandria	31,209188	29,884443	200	sand, *Caulerpa*	24	4
5	5	Eastern Harbour, Alexandria	31,207894	29,890154	200	*Caulerpa*, *Codium*, sand	29	5
6	6	Eastern Harbour, Alexandria	31,211206	29,901575	200	stony, *Caulerpa*	5	3
7	7	Close to Alexandria Harbour	31,198758	29,826388	200	small stones, *Caulerpa*	38	5
8	8	Close to Alexandria Harbour	31,189335	29,807257	200	small stones, *Caulerpa*, *Halimeda*	14	5
9	9	Western Harbour, Alexandria	31,162977	29,854056	200	mud with plants	14	2
10	10	Western Harbour, Port-Quest, Alexandria	31,176319	29,855383	200	*Caulerpa*, sand, mud	16	4
11	11	Western Harbour, Alexandria	31,191908	29,874419	200	smelling, black mud without algae	19	4
12	12	Western Harbour, Alexandria	31,183076	29,853813	200	*Caulerpa*, *Posidonia*, sand, some mud, *Codium* ground	14	3
13	13	Western Harbour, Alexandria	31,18791	29,867852	200	sand, some mud	34	2
14	14	Western Harbour, Alexandria	31,190708	29,859228	200	sandy, black mud with little *Caulerpa* and brown algae	11	1
15	15	Western Harbour, Alexandria	31,184171	29,862664	200	sand	8	2
16	17	Western Harbour, Port-Quest, Alexandria	31,168573	29,848745	200	little *Caulerpa*, sand, black mud	14	4
17	18	Western Harbour, Alexandria	31,165604	29,848059	200	very little *Caulerpa*, black mud	5	1
18	19	NW of Al Meks, Egypt	31,150471	29,835153	200	*Caulerpa*, sand	4	1
19	20	Off Al Meks, Egypt	31,150471	29,835153	200	*Posidonia*, *Cymodocea*	6	3
20	21	Near Dekheli, Egypt	31,137658	29,795965	200	*Caulerpa*, *Posidonia*, *Amphioxus*, brown algae	22	6
21	22	Great Pass, Alexandria	31,163205	29,82179	200	*Posidonia*, *Caulerpa*, brown algae, rocks, yellow sand	19	4
22	23	Margin of the Great-Pass, Alexandria	31,169944	29,810483	200	yellow *Amphioxus* sand, *Halimeda*, *Udotea*	10	5
23	24	Off Fort Ada	31,219602	29,876927	200	stony, *Caulerpa*, *Halimeda*	8	4
24	25	Infront of Fort Ada, Alexandria	31,207443	29,87276	350	*Caulerpa*, *Halimeda*, brown algae, seagrass	11	4
25	26	North of Al Max, Alexandria	31,386934	29,817017	200	soft and yellow mud with *Pteropodes*	11	3
26	27	North of El Anfushi Beach, Alexandria	31,36923	29,862703	200	mud with mollusc shells	27	5
27	28	Close to East Harbour, Alexandria	31,227026	29,905195	200	stones, *Caulerpa*, *Halimeda*	37	5
28	29	Close to East Harbour, Alexandria	31,221752	29,908885	200	stony, *Caulerpa*	6	3
29	30	Close to East Harbour, Alexandria	31,219315	29,910256	200	stones, *Caulerpa*	14	6
30	31	East Harbour, Port-Quest, Alexandria	31,203972	29,892629	200	sand, *Caulerpa*	14	3
31	32	Eastern Harbour, near entrance	31,210863	29,89358	200	coarse sand with stones and very little mud, *Caulerpa*	37	5
32	35	Off Sidi Bishr, Egypt	31,280191	29,960736	200	*Caulerpa*, *Posidonia*, *Halimeda*, rough sand with stones, *Amphioxus*, brown algae	51	6
33	36	Sidi Bischr	31,263725	29,976631	200	fine sand	10	3
34	37	Sidi Bischr	31,269151	29,971206	200	sandy, *Caulerpa*	4	2
35	38	NW of Abu Qir	31,340535	30,002044	200	*Halimeda*, *Caulerpa*, stony, brown algae	8	5
36	39	NW of Abu Qir	31,342819	30,025172	200	*Caulerpa*, sand, little mud	9	4
37	40	Abu Qir Bay	31,383222	30,173793	200	sand, stones, *Amphioxus* bottom	11	4
38	41	Crags at the eastern coast of Abu Qir Peninsula	31,323736	30,073142	200	crags with *Cystoseira*, *Padina*, little *Caulerpa*	15	4
39	42	Abu Qir, eastern coast	31,307793	30,082184	200	gray sand, mud, sea grass meadows	15	4
40	43	Western side of Abu Qir	31,326115	30,059817	200	crags, sand, brown algae, *Posidonia*, *Caulerpa*	8	3
41	44	Abu Qir Bay	31,473356	30,208724	200	fine rich slime	4	3
42	45	Abu Qir Bay	31,433191	30,2171	200	little sand, much mud	7	5
43	47	Abu Qir Bay	31,34753	30,232328	200	sandy with little mud	7	3
44	48	Lake Edku, seaside, sandy beach near the channel, in open water and upon stones of the bridge	31,273053	30,166655	200	shallow sand	27	3
45	49	Lake Edku, at the Bridge, near the canal with the Bay of Abu Qir, on shallow see-weed-bottom of the lake	31,254493	30,202109	800	shallow seagrass meadows	9	3
46	50	Off Abu Qir	31,359903	30,07219	200	sandy, stony, *Amphioxus* bottom	20	5
47	51	Abu Qir Bay	31,401544	30,082422	200	sandy ground, *Amphioxus* ground	18	4
48	52	North of Abu Qir	31,459032	30,065433	200	mud, *Phoronis* bottom	24	5
49	53	Off Abu Qir- Montazah	31,413346	29,992907	200	yellow mud, bottom with *Enteropneustes*	24	5
50	54	Northwest of Abu Qir Bay	31,456747	29,955787	200		17	4
51	55	Northwest of Abu Qir Bay	31,396785	29,963211	200	mud	25	4
52	56	Abu Qir Bay	31,343009	30,128584	200	coarse *Amphioxus* sand, little *Caulerpa*	9	4
53	57	Abu Qir Bay	31,331588	30,13715	200	fine sand, *Caulerpa*	21	3
54	58	Abu Qir Bay	31,307793	30,165227	200	sandy ground with algae (*Caulerpa*) and seaweed (*Cymodocea*)	23	4
55	60	North of Sidi Bishr	31,363948	29,950933	200	mud, bottom with *Enteropneustes*	8	4
56	61	North of East Port of Alexandria	31,390218	29,906104	200	stones, mud, algae (one empty shell)	60	5
57	62	North of Pharo, Alexandria	31,323117	29,931517	200	mud, *Caulerpa* bottom	27	5
58	63	NW of Agami island	31,313848	29,722266	200	stony coarse sand and mud	31	5
59	64	NW of Agami island	31,325667	29,710205	200	muddy ground mixed with coarse sand	23	6
60	65	North of Agamy	31,267858	29,790125	200	sand, mud, little *Caulerpa*	1	1
61	66	North of West Port of Alexandria	31,238162	29,853228	200	sand, mud, *Caulerpa*	18	3
62	67	North of West Port of Alexandria	31,274425	29,845804	200	sand, mud	22	4
63	68	North of West Port of Alexandria	31,328684	29,84538	200	*Caulerpa*, mud	14	4
64	69	North of West Port of Alexandria	31,345125	29,842823	200	*Caulerpa*, mud	15	4
65	71	North of East Port of Alexandria	31,275718	29,900393	200	stony, *Caulerpa*, *Halimeda*	2	2
66	72	North of Pharo, Alexandria	31,317692	29,894112	200	muddy, sandy, *Caulerpa*	16	2
67	73	North of East Port of Alexandria	31,352527	29,890114	200	*Caulerpa*, *Halimeda*, mud	13	5
68	74	North of Sidi Bishr	31,32226	29,964924	200	mud, *Caulerpa*, bottom with *Enteropneustes*	18	6
69	75	North of Pharo, Alexandria	31,305289	29,932008	200	sand, mud	12	4
70	76	Infront of East Harbour, Alexandria	31,220876	29,891905	200	stony, sandy, *Caulerpa*, *Halimeda*	15	4
71	77	North of Eastern Harbour, Alexandria	31,227577	29,884024	200	*Caulerpa*, stones, *Halimeda*	18	6
72	78	Close to West Harbour of Alexandria	31,202031	29,856423	200	*Caulerpa*, *Halimeda*, *Posidonia*, stony, brown algae	29	6
73	79	Close to West Harbour of Alexandria	31,20761	29,850944	200	*Caulerpa*, *Halimeda*, stony, rough sand	3	3
74	80	North-West of Harbour, Alexandria	31,211893	29,845233	200	sandy, stony, *Caulerpa*, *Halimeda*	1	1
75	81	Outer side of Fort El-Ayana	31,150983	29,784888	200	rocky *Amphioxus* sand, *Posidonia*	3	2
76	82	Outer side of Fort El-Ayana	31,146723	29,783032	200	*Halimeda* and brown algae	4	3
77	83	Inner side of the Fort el Ayana	31,14451	29,785435	200	crags with *Padina pav.*, shallow *Posidonia* bottom	6	2
78	84	Isle Agami, inside shoal beach	31,14708	29,789195	200	*Posidonia*, little *Caulerpa*, shoall beach with stones, brown algae	2	2
79	85	Isle Agami, inside	31,149531	29,796785	200	*Caulerpa*, *Posidonia*	4	3
80	86	Isle Agami, outside	31,154137	29,803791	200	coarse sand, *Amphioxus*, *Posidonia*, *Caulerpa*	4	4
81	87	Close to El Dukhaylah Beach, Egypt	31,146876	29,811992	200	*Caulerpa*, gray sand	3	2
82	90	North of Agami island	31,167349	29,78213	200	*Caulerpa*, mud	12	3
83	91	North of Agami island	31,198758	29,778132	200	muddy, *Caulerpa*, *Cymodocea*	7	2
84	92	North of Agami island	31,238162	29,778132	200	*Caulerpa*, mud	10	3
85	93	North of Port of El-Dekheila	31,159281	29,794416	200	coarse *Amphioxus* sand, *Caulerpa*, *Halimeda*, *Posidonia*	3	3
86	94	North of Port of El-Dekheila	31,160433	29,801688	200	stony, *Caulerpa*, *Halimeda*, *Sargassum*, *Posidonia*	7	4
87	95	North of Port of El-Dekheila	31,160486	29,806092	200	stony, *Caulerpa*, *Halimeda*, *Sargassum*, *Posidonia*	3	1
88	97	close to Port of El-Dekheila	31,159765	29,822968	200	*Caulerpa*, *Halimeda*, *Posidonia*, stony, *Amphioxus* bottom, brown algae	3	3
89	98	West of the Western Harbour of Alexandria	31,174891	29,840631	200	fine sand, *Posidonia*, *Caulerpa*	8	3
90	99	Close to Western Harbour of Alexandria	31,187071	29,841725	200	stones, sand, *Posidonia*, *Caulerpa*, *Halimeda*	6	3
91	100	West of the Western Harbour of Alexandria	31,178453	29,821961	200	*Caulerpa*, *Halimeda*, mussel-sand	9	2
92	101	Near Agami island	31,141831	29,772552	200	*Caulerpa*, *Posidonia*, *Halimeda*, brown algae, sand	7	3
93	102	Close to Agamy Beach	31,099868	29,725917	200	*Cystoseira* (brown algae), *Caulerpa*-*Halimeda* (green algae), stonny	18	3
94	103	North of Agamy Beach	31,14127	29,728202	200	*Caulerpa*, sand, mud	12	2
95	104	North of Agamy Beach	31,123853	29,726488	200	coarse sand, *Amphioxus* sand, *Halimeda*, *Caulerpa*	4	3
96	105	Close to Abu Talat, Egypt	31,053897	29,660244	200	dark sand, rotten, *Posidonia* with *Cystoseira*	11	3
97	106	Close to Abu Talat, Egypt	31,08245	29,658531	200	coarse sand	1	1
98	108	North of Abu Talat, Egypt	31,117571	29,660244	200	*Halimeda*, *Caulerpa*, brown algae, sand, stones	5	3
99	109	North of Abu Talat, Egypt	31,149836	29,665955	200	sand, stones, *Dasycladus*, *Halimeda*, *Cystoseira*	2	2
100	110	Near Agami island	31,134132	29,770613	200	crags with shallow sand bottom	2	1
101	111	Near Agami island	31,143174	29,76633	200	stony, *Caulerpa*, *Halimeda*, *Posidonia*	10	5
102	112	Near Agami island	31,149361	29,763475	200	*Caulerpa*, *Halimeda*, finer sand, little mud, *Amphioxus* bottom	7	4
103	113	Near Agami island	31,174488	29,733589	200	*Caulerpa*, sand, mud	3	3
104	114	North of Abu Talat, Egypt	31,176106	29,672808	200	stony, sand, mud, *Caulerpa*, *Halimeda*	20	4
105	115	North of Abu Talat, Egypt	31,19438	29,666526	200	stony, *Caulerpa*, *Halimeda*	7	4
106	116	North of Abu Talat, Egypt	31,219888	29,659635	200	*Caulerpa*, *Halimeda*, sand, mud, brown algae	12	5
107	117	North of Abu Talat, Egypt	31,257007	29,660777	200	mud, stones, *Caulerpa*, *Halimeda* sitting on calcareus algae	3	3
108	119	Great Pass, Alexandria	31,170344	29,820762	200	yellow sand, stones, mud, *Amphioxus* sand, *Caulerpa*, *Posidonia*	23	5
109	121	West of the Western Harbour of Alexandria	31,176454	29,833154	200	yellow coarse sand, stony, *Amphioxus* sand, *Caulerpa*, *Halimeda*	7	4
110	122	West of the Western Harbour of Alexandria	31,173256	29,827215	200	coarse sand, *Caulerpa*, *Amphioxus*	4	4
111	124	Close to Western Harbour of Alexandria	31,167546	29,841435	200	fine sand, *Caulerpa*	1	1
112	125	West of the Western Harbour of Alexandria	31,166974	29,825788	200	*Halimeda*, *Caulerpa*, yellow sand, stones, brown algae, *Amphioxus* bottom	32	5
113	126	West of the western Harbour of Alexandria	31,162749	29,832755	200	*Caulerpa*, *Posidonia*, dark sand	1	1
114	128	Port Al-Dikheila, Egypt	31,140061	29,803234	200	sand, mud, *Caulerpa*, *Posidonia*	2	2
115	134	East of Dekheila Port	31,148857	29,827965	200	*Posidonia*, *Caulerpa*, rough sand, little mud, *Amphioxus* bottom	8	3
116	135	Close to El Dukhaylah Beach	31,142015	29,825911	200	stony, sandy, *Caulerpa*, *Halimeda*, *Posidonia*	13	4
117	136	Close to El Dukhaylah Beach	31,14311	29,82553	200	*Caulerpa*, *Posidonia*, *Amphioxus* bottom	10	4
118	137	Close to El Dukhaylah Beach	31,139371	29,818676	200	dark sandy bottom, *Caulerpa*, little brown algae, *Cymodocea*	4	2
119	138	Close to El Dukhaylah Beach	31,140703	29,819652	200	*Caulerpa*, brown-algae, *Posidonia*, *Amphioxus* bottom	3	2
120	139	NW of Al Meks, Egypt	31,148629	29,832619	200	*Caulerpa*, *Posidonia*, rough sand	5	2
121	140	Entrance of Western Harbour, Alexandria	31,156752	29,841035	200	*Caulerpa*, *Posidonia*, stones, *Amphioxus* bottom, rough sand	19	5
122	141	Western Harbour, Port-Quest, Alexandria	31,173987	29,857049	200	sand, black mud	3	2
123	142	NW of Al Max	31,154798	29,831545	200	mud	1	1
124	143	North of Port of El-Dekheila	31,170407	29,799984	200	*Halimeda*, stony, *Amphioxus* bottom	6	3
125	144	North of Agami island	31,174202	29,791552	200	*Caulerpa*, *Halimeda*, rough sand, mud	9	4
126	145	NW of West Port of Alexandria	31,219734	29,833656	200	light gray mud, little *Caulerpa*	7	4
127	146	Off the Eastern Harbour, Alexandria	31,216802	29,89358	200	*Caulerpa*, *Halimeda*, *Posidonia*, *Dasycladus*	15	5
128	147	Nile mouth	31,453993	30,375344	2000	mud	8	2
129	148	Off Rosetta	31,397124	30,426265	1000	sandy	1	1
130	12IX	Lake Mareotis, near the Mex Experimental station or in open water	31,134285	29,855493	600	mud	4	2
131	13d	Western Harbour, arsenal basin	31,192419	29,863142	500	muddy, sand	1	1
132	14IX	Lake Mareotis, eastward, near the fresh-water fish-market, at the coast and towards the middle of the lake	31,168891	29,906109	1000	mud	4	2
133	25a	Infront of Fort Ada, Alexandria	31,209214	29,870289	200	*Amphioxus* sand, *Caulerpa*, *Cymodocea*	2	2
134	25c	Infront of Fort Ada, Alexandria	31,206832	29,873395	200	sand with *Cystoseira*, red algae, *Caulerpa*, *Ulva*	5	2
135	25d	Infront of Fort Ada, Alexandria	31,206085	29,874797	200	near the land sandy seagrass meadows, *Ulva*	4	2
136	28IX	Lake Mareotis, on the mole extending from the Mex Experimental station to the south, on floating sea weed and in open water and on stalks of reed in mud	31,113726	29,858768	1000	mud	12	4
137	33a	Head of the pier of Silsila, Alexandria	31,215089	29,904621	200	* Caulerpa *	4	3
138	33b	Off Silsila	31,212615	29,907514	200	sandy ground with *Cypraca*	9	3
139	34a	East Harbour, Silsila corner	31,20934	29,905116	200	shallow sandy, *Codium*, *Caulerpa*	18	4
140	34b	East Harbour, off Silsila	31,211206	29,903745	200	sand with *Caulerpa*	5	4
141	59a	North of Sidi Bishr	31,307127	29,977202	200	coarse sand, begrown with *Caulerpa* and *Halimeda*	6	3
142	59b	North of Sidi Bishr	31,299703	29,987196	200	coarse *Amphioxus* sand with algae	12	5
143	aquarium	Aquarium of Laboratory, Alexandria	31,212715	29,884665	200		2	1
144	BA	Off the Barracks of Ras el Tin, Po, Anfouchi Bay	31,205991	29,870975	200	Foraminifera sand in *Posidonia* meadows	1	1
145	coastAbuQir-near Rosetta	At the coast of the Bay of Abu Qir, washed ashore near Rosette	31,412646	30,34421	200	sand	8	3
146	D1	Lake Edku, near isle Derfil	31,269663	30,254223	200	black mud	10	3
147	D2	Lake Edku, near the village	31,294851	30,309155	600	mud, stones, sand	8	2
148	eastharbour1	Eastern Harbour: under the hull of a ship or epifauna or on the body of a ship or infront of bath and laboratory or Kayed Bay	31,208182	29,894361	1300	*Ulva* and coralline zone, *Caulerpa*	31	6
149	FortAda	Fort Ada	31,210875	29,875577	200	crags	5	1
150	L	Eastern Harbour, off the Laboratory,near the laboratory, before the laboratory	31,211614	29,884181	200	*Ulva*-coralline zone, rocks, *Caulerpa*	17	3
151	LacMarioutcenter	Lake Mayrut, in the middle of the lake, mud between the reeds on the bank	31,153046	29,898617	6000	mud	2	1
152	LakeEdku_marine beach	Lake Edku,marine beach	31,271933	30,17621	200	sandy beach	1	1
153	LakeEdkubridge	Lake Edku, bridge, under the stones and among sea weeds, near Edku Channel	31,267557	30,179252	400	mud, stones	4	1
154	nearbath	Eastern Harbour, near the bath, Alexandria	31,212364	29,885371	200	*Caulerpa*, stones, sand	24	4
155	offSidiBishr	Off Sidi Bishr	31,271107	29,98531	200		1	1
156	Pharo	Pharo, outerside or Kayed Bay outside in Kalkagen	31,2145	29,88479	200	calcareus algae	12	3
157	plankton1	Off Dekhela	31,170397	29,743697	200		6	3
158	plankton10	Plankton station X, Alexandria, at st. 26	31,386934	29,817017	200		2	2
159	plankton12b	Lake Edku, near isle Derfil	31,26878	30,253991	200	mud	1	1
160	plankton14	Plankton station XIV, Alexandria, at st54	31,456747	29,955787	200		2	1
161	plankton16	Plankton XVI, Alexandria, at st.64	31,325667	29,710205	200		2	2
162	plankton17	At st. 117, North of Abu Talat, Egypt	31,257007	29,660777	200	sandy, muddy, *Caulerpa*, *Halimeda*	2	2
163	plankton3	Off the Eastern Harbour	31,22687	29,892666	200		3	2
164	plankton4a	North of Abu Qir Bay	31,363663	30,016892	200	stony mud bottom	2	2
165	plankton6	Lake Mareotis, near the Mex Experimental station	31,143538	29,854457	1500		1	1
166	plankton7	Lake Mareotis, eastward, near the fresh-water fish-market, at the coast and further away from the coast in the plankton	31,178765	29,91436	1500		1	1
167	Po	Posidonia bottom, near the Ras El Tin Barracks	31,204189	29,869984	200	* Posidonia *	6	3
168	POK	Crags outside the Barracks of Ras El Tin, off the posidonia bottom	31,207036	29,86802	200	crags, *Caulerpa*, *Halimeda*, brown algae	22	4
169	SidiBishr	Sidi Bishr	31,26592	29,98614	500	zone of algae on rocks	18	3
170	Silsila	Beach of Silsila	31,211061	29,909446	200	sand	1	1
171	Trawl(T)	Trawl in Abu Qir Bay	31,427605	30,23248	6000	muddy ground with algae	16	4
172	westernharbour1	Western Harbour, Arsenal Basin, West Harbour epifauna	31,194964	29,874831	800		7	3

**Table 3.** List of the most common species found in more than 10% of the stations

**Scientific Name**	**Scientific Name accepted**	**Kingdom**	**Phylum**	**Class**	**Order**	**Family**	**Number of stations**
*Dentalium (Antalis) dentale forma inaequicostatum*	*Antalis inaequicostata*	Animalia	Mollusca	Scaphopoda	Dentaliida	Dentaliidae	41
*Branchiostoma lanceolatum*	*Branchiostoma lanceolatum*	Animalia	Chordata	Leptocardii		Branchiostomatidae	25
*Chlamys glabra*	*Flexopecten glaber*	Animalia	Mollusca	Bivalvia	Pectinida	Pectinidae	21
*Pilumnus hirtellus*	*Pilumnus hirtellus*	Animalia	Arthropoda	Malacostraca	Decapoda	Pilumnidae	21
*Columbella rustica*	*Columbella rustica*	Animalia	Mollusca	Gastropoda	Neogastropoda	Columbellidae	19
*Abra ovata*	*Abra segmentum*	Animalia	Mollusca	Bivalvia	Cardiida	Semelidae	19
*Glycymeris pilosus var lineatus*	*Glycymeris bimaculata*	Animalia	Mollusca	Bivalvia	Arcida	Glycymerididae	19
Beguina (Glans) trapezia	*Glans trapezia*	Animalia	Mollusca	Bivalvia	Carditida	Carditidae	17
Murex (Truncularia) trunculus	*Hexaplex trunculus*	Animalia	Mollusca	Gastropoda	Neogastropoda	Muricidae	17
Nassarius (Hinia) incrassatus	*Tritia incrassata*	Animalia	Mollusca	Gastropoda	Neogastropoda	Nassariidae	17

**Table 4.** Column description of 'Darwin Core Event' table

**Column label**	**description**
id	Same as eventID
type	The nature or genre of the resource (dataset etc)
eventID	An identifier for the sampling event (event=sampling that occurs at a place,time, specific protocol and depth)
parentEventID	An identifier for the broader Event at a specific station, that groups this and potentially other Events
samplingProtocol	The name of, reference to, or description of the method or protocol used during an Event
eventDate	The date-time or interval during which an Event occurred
year	The four-digit year in which the Event occurred, according to the Common Era Calendar
month	The original month in which the Event occurred
day	The original day in which the Event occurred
habitat	A category or description of the habitat in which the Event occurred
fieldNumber	The sample name
eventRemarks	Comments or notes about the Event
locationID	Station name
locality	The name or description of the place
minimumDepthInMeters	The lesser sampling depth of a range of depth below the local surface, in meters
maximumDepthInMeters	The greater sampling depth of a range of depth below the local surface, in meters
locationRemarks	Comments or notes about the station where the sample occurred
decimalLatitude	The geographic latitude (in decimal degrees) of the station
decimalLongitude	The geographic longitude (in decimal degrees) of the station
coordinateUncertaintyInMeters	The horizontal distance (in meters) from the given decimalLatitude and decimalLongitude describing the smallest circle containing the whole of the Location

**Table 5.** Column description of 'Darwin Core Occurrence' table

**Column label**	**description**
id	Same as occurrenceID
type	The nature of the resource (e.g. dataset)
institutionCode	The name in use by the institution having custody of the object(s) or information referred to in the record
collectionCode	The name, coden, or initialism identifying the collection or data set from which the record was derived
basisOfRecord	The specific nature of the data record
occurrenceID	A unique identifier for the Occurrence
catalogNumber	An identifier for the record within the data set or collection
occurrenceRemarks	Comments or notes about the Occurrence
individualCount	The number of individuals represented present at the time of the Occurrence
sex	The sex of the biological individual(s) represented in the Occurrence
lifeStage	The age class or life stage of the biological individual(s) at the time the Occurrence was recorded
eventID	An identifier for the sampling event (event=sampling that occurs at a place,time, specific protocol and depth)
identifiedBy	A list of names of people, groups, or organizations who assigned the Taxon to the subject
identificationReferences	A list of references (publication, global unique identifier, URI) used in the Identification
scientificNameID	The lsid from WORMS
scientificName	The full scientific name. When forming part of an Identification, this should be the name in lowest level taxonomic rank that can be determined
kingdom	The full scientific name of the kingdom in which the taxon is classified
phylum	The full scientific name of the phylum or division in which the taxon is classified
class	The full scientific name of the class in which the taxon is classified
order	The full scientific name of the order in which the taxon is classified
family	The full scientific name of the family in which the taxon is classified
genus	The full scientific name of the genus in which the taxon is classified
subgenus	The full scientific name of the subgenus in which the taxon is classified. Values should include the genus to avoid homonym confusion
specificEpithet	The name of the first or species epithet of the scientificName
scientificNameAuthorship	The authorship information for the scientificName formatted according to the conventions of the applicable nomenclaturalCode
nomenclaturalCode	The nomenclatural code under which the scientificName is constructed
taxonRemarks	Comments or notes about the taxon or name

**Table 6.** Column description of 'Darwin Core Extended Measurement or Facts' table

**Column label**	**description**
eventID	An identifier for the sampling event (event=sampling that occurs at a place, time, specific protocol and depth)
measurementID	An identifier for the MeasurementOrFact
measurementType	The nature of the measurement, fact, characteristic, or assertion
measurementValue	The value of the measurement, fact, characteristic, or assertion
measurementUnit	The units associated with the measurementValue
occurrenceID	A unique identifier for the Occurrence
measurementMethod	A description of or reference to (publication, URI) the method or protocol used to determine the measurement, fact, characteristic, or assertion
measurementDeterminedBy	A list of names of people, groups, or organizations who determined the value of the MeasurementOrFact
measurementTypeID	An identifier for the measurementType (global unique identifier, URI)
measurementAccuracy	The description of the potential error associated with the measurementValue
measurementRemarks	Comments or notes accompanying the MeasurementOrFact
